# A two-centre experience of tonsil biopsies in the investigation of patients with tonsillar asymmetry

**DOI:** 10.1308/rcsann.2022.0122

**Published:** 2023-01-23

**Authors:** E Campbell, O McLaren, A Sheldon, B Rock, TS Bracey, T Malik, VM Reddy

**Affiliations:** ^1^ Royal Cornwall Hospital NHS Trust, UK; ^2^ University Hospitals Plymouth NHS Trust, UK

**Keywords:** Pathology, Biopsy, Tonsil, Cancer, Tonsillectomy

## Abstract

**Introduction:**

We aim to evaluate our experience of tonsil biopsies in the investigation of patients presenting with asymmetrical tonsils.

**Methods:**

A two-centre retrospective analysis of all patients who underwent histology sampling of the palatine tonsils between 1 January 2013 and 31 December 2018 was completed. Data collected included patient demographics, method of obtaining tonsil tissue, histological diagnosis and need for repeat tissue sampling. A follow-up period of 36 months was allowed to establish whether any patients re-presented with missed diagnoses.

**Results:**

In total, 937 patients were included for analysis: 375 (40.0%) had a biopsy, of which 191 (50.9%) were performed in clinic. The mean duration from initial appointment with the ear, nose and throat clinic to tissue sample collection was 17.6 days (range 0–327 days) for all biopsies, reducing to 0.2 days (range 0–17 days) for biopsies performed in clinic. This was significantly shorter than for tonsillectomies (mean 38.9 days, range 0–444 days; *p*<0.05). Of the patients who underwent tonsil biopsy, six (1.6%) had malignancy that was not unequivocally diagnosed on initial biopsy. In all six patients, prior clinical suspicion was high, and repeat tissue sampling was undertaken on receipt of negative histology results.

**Conclusions:**

Tonsil biopsy is a viable alternative to tonsillectomy for histology in the assessment of tonsil asymmetry. Tonsil biopsy in the outpatient setting has reduced surgical morbidity, significantly less delay in diagnosis, less inconvenience for patients and lower healthcare costs compared with formal tonsillectomy. Although tonsil biopsies should not be used in isolation, they can be useful in the investigation of patients presenting with tonsillar asymmetry.

## Introduction

Patients present to ear, nose and throat (ENT) clinics with asymmetrical palatine tonsils without other symptoms, or in association with pathological cervical lymphadenopathy, or incidentally found on examination or imaging. Traditionally, such cases are investigated by tonsillectomy for histological diagnosis.

The rate of malignancy in asymmetrical tonsils without associated signs and symptoms of malignancy is negligible.^1-4^ Tonsil biopsy offers a means of obtaining histological diagnosis without a general anaesthetic, preoperative assessment, and the morbidity of tonsillectomy including pain and bleeding.^5^

The option of tonsil biopsy has been standard practice in our departments (which collaborate as a joint head and neck multidisciplinary team) for several years. Tonsil biopsy is performed at the discretion of the clinician under local anaesthetic in the outpatient department for patients with asymmetry or focal abnormality. In our experience, this approach facilitates earlier diagnosis compared with tonsillectomy without associated morbidity.

The aim of this retrospective service evaluation is to assess the usage of tonsil biopsy in our network and quantify delays to tissue sampling compared with tonsillectomy.

### Tonsil biopsy technique in the outpatient setting

Patients are typically seen in the head and neck suspected cancer clinic, which incorporates a one-stop neck lump clinic. We have established that each clinician (consultant, associate specialist, staff grade and/or specialist registrar) would be allocated seven patients, with 25-minute slots per patient. Through a process of trial and error, we identified this as the ideal clinic template to allow timely running of the clinic while allowing discussion of ultrasound results, taking consent for future procedures, undertaking all administrative tasks and performing biopsies under local anaesthetic (oropharyngeal and laryngeal) where appropriate. In cases in which it is not clear whether a tonsil biopsy is appropriate, this can be discussed with the consultant leading the clinic session.

Once a patient has agreed to proceed to tonsil biopsy in clinic, written consent is obtained having explained the procedure and risks including infection, bleeding and failure to make a diagnosis, which may require further investigation or procedure. The World Health Organization surgical safety checklist is completed. The oropharynx is inspected using a headlight and metal tongue depressor. Approximately five sprays of xylocaine 10mg anaesthetic spray are applied to the oropharynx on the side to be biopsied because this helps to minimise the gag reflex. After 1min, the oropharynx is again inspected with the tongue depressed. A single-use cervical rotating biopsy punch (DTR Medical Ltd, Swansea, UK) is used to obtain a biopsy of the tonsil which is then sent for histology. The oropharynx is reinspected to ensure there is no significant ongoing bleeding, which can be managed with bipolar cautery, prior to the patient leaving the outpatients department. The patient is advised to remain nil by mouth until the local anaesthetic has worn off.

## Methods

A two-centre service evaluation was undertaken involving a retrospective case note review of palatine tonsil biopsy and tonsillectomy for histology between 1 January 2013 and 31 December 2018 inclusive. The study period allowed a minimum follow-up period of 36 months in which patients may have presented again with missed diagnoses or needed further investigations.

Cases were identified by interrogating hospital pathology information systems for the presence of the terms ‘tonsil’, ‘tonsillectomy’ or ‘oropharynx’ in the histology report. The histology reports and electronic case notes were reviewed to identify only those cases relating to palatine tonsils.

Cases were excluded where the clinical scenario would preclude tonsil biopsy; carcinoma of unknown primary cases because the tonsils would look unremarkable; and tonsil sampling as a secondary procedure to other operations such as neck dissection or branchial cyst excision. Patients were excluded if the decision to send tonsils for histology was made on the day of surgery; typically these were patients with recurrent tonsillitis who had asymmetrical tonsils noted at time of tonsillectomy.

Data collected included patient demographics, date of initial presentation, indication for biopsy, nature of procedure, date of specimen collection and final histological diagnosis. It was also noted whether patients required a further procedure, either as part of their initial presentation or following re-referral.

### Statistical analysis

Data were recorded on an Excel spreadsheet (Microsoft Corp., Redmond, US), which was used to produce descriptive statistics. The delay from initial ENT consultation to tissue sampling was compared with the Mann–Whitney *U* test using IBM SPSS Statistics for Windows, version 28 (IBM Corp., Armonk, US). The rate of success and failure of tonsil biopsy was established, with failure being defined as the need to repeat the tonsil biopsy or proceed to formal tonsillectomy for histological diagnosis. We also explored the variation in techniques over time (tonsil biopsy in clinic, tonsil biopsy under general anaesthetic and tonsillectomy).

The project was registered with institutional audit and quality improvement departments. Because this was a retrospective service evaluation ethical approval was not required.

## Results

From 2014 to 2018, 984 adult patients were identified who had samples of palatine tonsils taken for histological diagnosis. Forty-seven patients were excluded because clinical information was incomplete (4.4%).

Some 937 patients were included for analysis, consisting of 485 males (mean age 49.0 years, range 16–87 years) and 452 females (mean age 47.1 years, range 16–90 years).

Of the patients in the study, 375 (40.0%) had a biopsy, of which 191 (50.9%) were performed under topical anaesthesia in clinic and 182 (48.5%) were performed under general anaesthesia in theatre. Some 562 patients had a formal tonsillectomy. The number of biopsies vs tonsillectomies over time is demonstrated in Table 1. The histology results for all patients are shown in Table 2.

**Table 1:** Frequency of diagnostic tonsillectomies and tonsil biopsies by year

	**2013**	**2014**	**2015**	**2016**	**2017**	**2018**
Total tonsillectomies and tonsil biopsies sent for histology (*n*)	87	160	181	182	166	161
Tonsillectomies (*n*) Percentage of total sent for histology	64 73.6	107 66.9	116 64.1	107 58.8	76 45.8	92 57.1
Tonsil biopsies (in theatre or in clinic) (*n*) Percentage of total sent for histology	23 26.4	53 33.1	65 35.9	75 41.2	90 54.2	69 42.9
Tonsil biopsies performed in theatre (*n*) Percentage of all tonsil biopsies	19 82.6	27 50.9	38 58.5	41 54.7	31 34.4	28 40.6
Tonsil biopsies performed in clinic (*n*) Percentage of all tonsil biopsies	4 17.4	26 49.1	27 41.5	34 45.3	59 65.6	41 59.4

**Table 2:** Histological diagnoses in patients undergoing tonsillectomy (*N*=562) and biopsy (*N*=375)

**Diagnosis**	**Tonsillectomy** *** n* (%)**	**Biopsy ** ***n* (%)**
Normal/reactive	477 (84.9)	145 (38.7)
Benign lesion	20 (3.6)	101 (26.9)
SCC p16 positive	40 (7.1)	65 (17.3)
SCC p16 negative	6 (1.1)	13 (3.5)
SCC p16 not known	4 (0.7)	22 (5.8)
Lymphoma	10 (1.8)	24 (6.4)
Other malignancy	4 (0.7)	2 (0.5)
Dysplasia	1 (0.2)	3 (0.8)

SCC = Squamous Cell Carcinoma

The mean duration from initial appointment with ENT to tissue sample collection was 17.6 days (range 0–327 days, median 0 days) for all biopsies performed in clinic or theatre, reducing to 0.2 days (range 0–17 days, median 0 days) for biopsies performed in clinic. For tonsillectomies, the mean duration was 38.9 days (range 0–444 days, median 28 days).

The median duration between clinic appointment and collection of tissue sample for tonsillectomy was 28 days, compared with a median of 0 days for tonsil biopsy. Mann–Whitney *U* test demonstrated a statistically significant difference in time from initial consultation to tissue sampling between patients undergoing a tonsillectomy and those who had a biopsy (*p<*0.01).

Three patients waited more than 300 days from when they were seen in clinic to their histology being reported. On further interrogation of the notes, these patients were felt to have benign pathology when seen in clinic and were therefore listed routinely for procedures under general anaesthetic.

Of the 375 patients who underwent a biopsy, 14 (3.7%) underwent a further procedure to obtain tissue for histology.

• Ten patients required a further procedure to aid in diagnostics, six of whom were subsequently diagnosed with malignancy (see below):
• Two patients diagnosed with Squamous Cell Carcinoma (SCC) on biopsy underwent treatment, then subsequently underwent further panendoscopy with tonsil biopsies owing to a concern regarding recurrence found at surveillance follow-up;
• two patients re-presented to ENT 2 years after being diagnosed with benign tonsillar asymmetry on biopsy, repeat biopsy showed no malignancy.

• Six patients (1.6%) who underwent biopsy were later diagnosed with malignancy that was not found during initial biopsy:
• two were symptomatic of lymphoma with normal tonsil biopsies and diagnosis was made on subsequent core biopsy of cervical lymph nodes;
• one had lymphoma in repeat tonsil biopsies taken in theatre;
• one had mucoepidermoid carcinoma diagnosed on tonsillectomy, with earlier clinic biopsy showing features in keeping with a mucocele, although it was noted at the time that this was a technically difficult procedure that was poorly tolerated, with the clinician indicating that the sample may not be representative;
• one had a clinically suspicious tonsil lesion with a negative tonsil biopsy in clinic, and underwent further tonsil biopsies under general anaesthetic which confirmed SCC;
• one had a tonsil biopsy in clinic reported as dysplasia, diagnosed as p16-positive SCC following tonsillectomy. On subsequent MDT review by the head and neck specialist lead histopathologist, the initial tonsil biopsy was shown to demonstrate p16-positive SCC rather than dysplasia as initially reported.

No patients discharged from ENT later re-presented with a missed diagnosis of cancer.

## Discussion

We have reported our experience of 937 patients across two sites who had tonsil tissue sent for histology over a 6-year period. Some 375 patients underwent a tonsil biopsy, 191 of which were performed in clinic; 562 patients had a tonsillectomy. The patients who underwent a tonsil biopsy would have received a tissue diagnosis significantly faster than those who had a tonsillectomy, more so for those patients who had a tonsil biopsy performed in clinic rather than in theatre. Our data demonstrate that the proportion of patients having a biopsy instead of tonsillectomy has increased over time.

Of 375 patients who underwent a tonsil biopsy, only 6 (1.6%) had malignancy diagnosed on subsequent histology. In all these cases there was a negative tonsil biopsy despite a high index of suspicion of malignancy prompting further investigation, as detailed earlier.

The involvement of specialist head and neck pathologists is preferable because they are more aware of some of the peculiar diagnostic pitfalls in the diagnosis of Human Papilloma Virus (HPV)-related oropharyngeal SCC. For example, the non-infiltrative pushing pattern of invasion in small biopsies is often misinterpreted as dysplasia by a less experienced generalist.^6^

The results of tonsil biopsy should not be viewed in isolation, however. Clinical judgement must be applied in conjunction with histology results to establish whether further investigation is warranted. Any patient who had a malignancy that was not diagnosed on biopsy was later diagnosed on tonsillectomy or further biopsy under a general anaesthetic. The combined approach of clinical judgement and tonsil biopsy appears to be successful as, importantly, no patients were discharged from ENT following a negative biopsy who then re-presented with a malignancy.

The rate of malignancy in unilateral enlarged tonsils without associated symptoms is negligible. If there are associated symptoms, and high clinical suspicion, the rate for malignancy is 5%–9%.^1-4^ This would support our proposition that tonsil biopsies are a safe and effective tool that can be particularly useful in the following situations:

• In patients with asymmetrical tonsils, no concerning symptoms and low clinical suspicion, a negative biopsy can be useful to reassure and discharge the patient.
• Patients diagnosed with malignancy on tonsil biopsy where the primary site is too extensive for surgical management will benefit from immediate referral for oncological treatment following radiological staging and multidisciplinary team discussion.

Patients in whom the clinician has a high level of clinical suspicion for malignancy, and either suspected to have an unrepresentative sample, or a negative tonsil biopsy result, would be offered formal tonsillectomy for histology.

We do not advocate the use of tonsil biopsies in patients with an SCC of unknown primary. Waltonen *et al*^7^ found that occult tonsil malignancies are more likely to be diagnosed on tonsillectomy (29.6%) than tonsil biopsy (3.2%), and one patient in that study was subsequently diagnosed with an SCC that had been missed on tonsil biopsy.

We have not considered the role of examination under anaesthesia of a presumed oropharyngeal primary to assess suitability for transoral laser or robotic surgery, which is beyond the remit of this service evaluation. Historically, it has been suggested that tonsil biopsy may impair subsequent staging imaging; it is the opinion of the radiologists at our institutions that this is rarely an issue provided the reporting radiologist is made aware of the timing of biopsy.

### Study limitations

Limitations of our service evaluation include the reliance on retrospective data that were not standardised. The data for waiting time for histology results in patients who underwent tonsillectomy may be skewed by patients who were viewed as low or no clinical risk of malignancy and therefore were not booked urgently, although these are small numbers that do not impact on the overall conclusions. Although there were no patients re-presenting to our ENT departments with a missed malignancy, this service evaluation does not account for patients who may have moved out of the region and presented to ENT departments elsewhere and who we may not have been made aware of.

## Conclusions

Tonsil biopsy is a viable method for obtaining tonsil tissue for diagnosis. Tonsil biopsies represent significantly less waiting time for histological diagnosis, less inconvenience for patients (especially if performed in the clinic under a local anaesthetic) and less overall cost. Although tonsil biopsies should not be used in isolation from clinical judgement, they are particularly useful in the investigation and reassurance of patients presenting with tonsillar asymmetry. The corroboration of benign pathology in the absence of clinical suspicion is of value to patients.

The response to the coronavirus pandemic has impacted on the availability of operating theatre capacity leading to longer waiting times for surgery. In this climate, clinic-based diagnostic procedures are particularly appealing. The utilisation of tonsil biopsies more widely could help to reduce the burden on waiting times for surgery and expedite patient care.

In appropriate clinical scenarios involving focal irregularity or gross asymmetry of palatine tonsils, biopsy in the outpatient setting is favourable compared with formal tonsillectomy in terms of reduced surgical morbidity, quicker diagnosis and lower healthcare costs. This can facilitate earlier diagnosis and delivery of definitive treatment.
